# Dynamic Microbial Shifts and Signatures of Long-Term Remission in Allergic Rhinitis After an Herbal Formula Treatment

**DOI:** 10.3389/fimmu.2021.774966

**Published:** 2021-10-22

**Authors:** Libing Zhu, Yuning Wu, Chenglong Lin, Lin Tang, Bin Yu, Wenrong Wan, Jingxiu Xuan, Yanling Du, Zhangran Chen, Wei Liang

**Affiliations:** ^1^ Department of Traditional Chinese Medicine, School of Medicine, Xiamen University, Xiamen, China; ^2^ Department of Traditional Chinese Medicine, Xiamen University Hospital, Xiamen, China; ^3^ School of Mathematical Sciences, Xiamen University, Xiamen, China; ^4^ Department of Otorhinolaryngology, Xiamen Hospital of Traditional Chinese Medicine, Xiamen, China; ^5^ Internal Medicine Department of Traditional Chinese Medicine, Xiamen Hospital of Traditional Chinese Medicine, Xiamen, China; ^6^ Laboratory of Rheumatology and Immunology, The First Affiliated Hospital of Xiamen University, Xiamen, China; ^7^ Department of Acupuncture and Tuina, Fujian University of Traditional Chinese Medicine, Fuzhou, China; ^8^ Institute for Microbial Ecology, School of Medicine, Xiamen University, Xiamen, China

**Keywords:** Xiao-Qing-Long-Decoction, allergic rhinitis, long-term remission, gut microbiota, 16S rRNA

## Abstract

A mixed Chinese herbal formula, Xiao-Qing-Long-Decoction (XQLD), may contribute to sustained remission in allergic rhinitis (AR), but it is unknown which factors determine such long-term effect. Here, we aimed to identify bacterial signatures associated with sustained remission. To this end, samples from AR patients at four different times were analyzed to compare the dynamic bacterial community and structure shifts. Diversity indices Chao1 showed significant difference across different time (*p*<0.05), and the Kruskal-Wallis test identified that *Dialister* (OTU_31), *Roseburia* (OTU_36), *Bacteroides* (OTU_22), *Bacteroides* (OTU_2040), and *Prevotella_*9 (OTU_5) were the significant differential bacterial taxa (*p*<0.05). These distinctive genera were significantly associated with the change of AR clinical indices and the predicted functional pathways such as PPAR signaling pathway, peroxisome, and citrate cycle (TCA cycle) (*p*<0.05), indicating that they may be important bacterial signatures involving in the sustained remission in AR (*p*<0.05). Besides, lower Firmicutes/Bacteroidetes (F/B) ratio at 6 months follow-up may also contribute to the long-term remission of AR. No seriously adverse events and safety concerns were observed in this study. In conclusion, XQLD is a meaningful, long-term efficient and safe medication for AR treatment. The underlying mechanisms of sustained remission in AR after XQLD treatment may be associated with the dynamic alteration of featured gut bacteria taxa.

## Introduction

The complex ecosystem of the commensal microbiota and its interactions with the host are fundamental in determining health (1). Gut microbiota imbalance has been associated with allergic disease, including allergic rhinitis (AR) (2, 3). AR is characterized as an immunoglobulin E (IgE)–mediated inflammatory response of the nasal mucous membranes after exposure to inhaled allergens, which is estimated to affect nearly one in every six Americans and generates $2 to $5 billion in direct health expenditures annually (4, 5). Our previous study has reported that AR patients had a higher abundance of Firmicutes, which have been reported to be associated with the development of AR in early childhood (4, 5). Altered microbial diversity in early infancy precedes the development of AR and asthma at school age (6–8). The increase of serum total immunoglobulin E (tIgE) level is a risk factor for allergen sensitization in children, and some studies have found that the decrease of gut microbiota diversity can be linked with the increasing of serum tIgE level (9). Taken together, these studies indicated that microbiota dysbiosis may contribute to AR development, serving as a potential new target for disease control.

To treat AR, allergic asthma, and other allergic disease, several attempts have been made *via* modulating the gut microbiota (10). A Chinese herbal formula, Formula-3, showed to ameliorate food allergy (FA) partially *via* restoring the bacterial balance by increasing Bacteroidetes while decreasing Firmicutes abundance (11). Another Chinese herbal formula, Dongbing Xiazhi Fang, has proven to be efficient in treating asthma, perhaps through regulating the gut microbiota in asthmatic guinea pigs (12). Hence, Traditional Chinese Medicine (TCM) may serve as a new source of drugs for gut microbiota-targeted disease management.

Xiao-Qing-Long-Decoction (XQLD), also known as So-Chenog-Ryong-Tang in Korea and Sho-seiryu-to in Japan, is a mixed Chinese herbal formula that has been commonly used for AR in China (13). The use of XQLD was firstly recorded in Shang Han Lun by the prestigious physician Zhongjing Zhang (AD 150–219), which has been reported to have potentially beneficial effects in the treatment of AR in animal trials, as well as in some clinical observations (14–16). A recent animal study revealed that XQLD reduce the inflammation in heart failure with preserved ejection faction (HFpEF) model rats by regulating the composition of the gut microbiota (17). Besides, recent evidence has indicated that XQLD influenced gastrointestinal motility (18). Although these findings indicate that the gut microbiota might have a pivotal role in the effect of XQLD on AR subjects, yet there is still a lack of direct evidence. Consequently, we conduct this study to identify specific bacterial groups that are signatures and predictors for a sustained response to XQLD therapy, attempting to elaborate the action mechanisms of XQLD from the perspective of human microbiota.

## Materials and Methods

### Study Design

The study was a case series study with 10-week treatment and 24-week follow-up that was approved by the Ethics Committee of Xiamen Hospital of Traditional Chinese Medicine (Permit Number ID: 2019-K020-01), and later it was registered in Chinese Clinical Trial Registry (ChiCTR1900028613). Participants were recruited by Xiamen Hospital of Traditional Chinese Medicine (Xiamen, China) or Xiamen University Hospital (Xiamen, China) from December 2019 to September 2020. All participants signed informed consent forms before beginning the study. The study was conducted in accordance with the principles of the Declaration of Helsinki (19).

Recruitment was undertaken *via* screening patients who randomly visit the authors’ (LZ) TCM clinic or (LT and BY) otorhinolaryngology clinic. The inclusion and exclusion criteria for AR subjects were the same as the previous study (2). Treatment was delivered in the usual setting of the authors’ (LZ) TCM clinic in Xiamen University Hospital (Xiamen, China). Fifty-three diagnosed AR patients who had not received prior pharmacologic treatment for AR were recruited into the study. After the study was completed, a total of 33 patients were included for final analysis (**Figure S1** and **Table S1**).

### Drug Administration

The TCM formula in our study was XQLD, composed of eight herbs, namely, Zingiber officinale Roscoe, Cinnamomum cassia Blume, Ephedra sinica Stapf, Paeonia lactiflora Pall., Glycyrrhiza uralensis Fischer, Asiasarum sieboldi F. Maekawa, Pinellia ternata Breitenbach, and Schisandra chinensis (Turcz.) Baillon (**Table S2**). Herbs were all provided and quality controlled by Good Manufacturing Practice (GMP)-certified pharmaceutical company Beijing Tongrentang. The TCM intervention was given as decoction. They were prepared by Chinese pharmacy of Xiamen University Hospital according to a standard production process. Each unit of XQLD formula yielded 300 ml of decoction. Each patient orally took 150 ml of the decoction two times daily for 10 weeks. All the decoctions were quality controlled throughout the study. Each subject was seen by the registered TCM practitioner (LZ), and a full case history was taken, including tongue and pulse observations. Subsequent consultations were held once a week (again reflecting normal practice).

### Study Evaluation and Outcomes

The following primary efficacy outcomes were used: changes in Total Nasal Symptom Score (TNSS) and gut microbiota. Secondary efficacy outcomes included changes in Rhinoconjunctivitis Quality of Life Questionnaire (RQLQ), eosinophil (EO) counts, cytokines [interleukin-2 (IL-2), tumor necrosis factor-alpha (TNF-α), interferon-gamma-γ (IFN-γ), IL-4, IL-6, and IL-10], and tIgE. The TNSS is calculated based on the sum up of nasal obstruction, rhinorrhea, nasal itching, and sneezing, which represents perspectives of AR symptom, that is, the higher score means the worse symptom condition. RQLQ score, which consists of activity limitation, sleep disorders, non-eye/nasal symptoms, practical problem, nasal symptom, eye symptoms, and emotions, reflects the life quality status that was associated with AR. The value for each score and RQLQ positively correlated with the extent of the AR impact on the life quality. The safety evaluation included the levels of aspartate aminotransferase (AST), alanine aminotransferase (ALT), blood urea nitrogen (BUN), creatinine, routine urine test, and complete blood counts (CBC). All study assessments were performed at 0, 10, 14, and 34 weeks. Registered TCM Practitioner (LZ) asked participants questions regarding adverse events (AEs) during every visit and recorded AE details in case report forms.

### Clinical and Biochemical Measurements

Biochemical measurements of AST, ALT, BUN, creatinine, routine urine test, and CBC were performed in the laboratory of Xiamen University Hospital. Cytokines including IL-2, TNF-α, IFN-γ, IL-4, IL-6, IL-10 were performed in the laboratory of hematology department (the First Affiliated Hospital of Xiamen University, Xiamen, China); tIgE was performed in the laboratory of rheumatology and immunology department (the First Affiliated Hospital of Xiamen University, Xiamen, China). Cytokines were measured by immunofluorescence technique (BD FACScalibur flow cytometry, USA). ELISA Kits were used to measure tIgE (R&D System, USA). Automatic hematology analyzer was used to measure CBC (Mindray automatic hematology analyzer BC-20, China). The urine was detected and analyzed by dry chemical test strip (SYSMEX automatic urine analyzer UC-3500, Japan). AST, ALT, BUN, and creatinine were measured by automatic biochemical analyzer (SYSMEX BX-4000, Japan).

### Fecal DNA Extraction and 16S rRNA Gene Sequencing

Fecal samples from AR patients were collected on the day of the medical examination and immediately frozen at −80°C before use. Fecal DNA (from approximately 0.25 g subsamples) was extracted using the QIAamp Fast DNA Stool Mini Kit (Qiagen, CA, USA). The purity and concentration of the isolated DNAs were assessed using spectrophotometry (MultiskanTM GO, Thermo Fisher Scientific, USA). The V3-V4 hypervariable region of the bacterial 16S ribosomal RNA (rRNA) gene was amplified from the DNA samples with the barcoded forward primers 341F (5’-CCTACGGGNBGCASCAG-3’) and the reverse primers 806R (5’-GGACTACNVGGGTWTCTAAT-3’) using KAPAHIFI HotStart ReadyMix (KAPA Biosystems, USA). The raw paired-end reads were assembled using the flash (20). The chimera checking and OTU clustering were performed with the clean tags by usearch (21). In details, all reads were demultiplexed into one file, clustered at 97% similarity, then the chimera checking was performed using UCHIME (21) in reference mode. Representative sequences were generated, singletons were removed, and a final OTU table was created. The representative sequences of OTU were aligned on the Greengenes (22) database for taxonomic classification by RDP Classifier (23).

### Statistical Analysis

The significantly distinguished TNSS, RQLQ, tIgE, EO counts, percentage of EO, cytokines, taxa, and predicted pathway by PICRUSt (24) were screened by comparison among groups by Kruskal-Wallis test (*p*<0.05). Bacterial alpha diversity, community ordination based on Bray-Curtis distance, PERMANOVA and ANOSIM analysis were based on the R package vegan (version 2.5.5) (25). Non-metric multidimensional scaling (NMDS) plot based on Bray–Curtis distances calculated using taxa compositions was conducted with the metaMDS function and graphed by ggplot2 (26, 27). Principal coordinate analysis (PCoA) was performed using the package ape. The shared and unique OTUs were calculated and visualized using the R package VennDiagram (version 1.6.20) (28). Spearman’s correlations test between the abundances of differential genera taxa and TNSS, RQLQ, tIgE, EO counts, percentage of EO, or cytokines were computed by the R package stats (version 3.6.0), and package pheatmap (version 1.0.12) were used to conduct the correlation heatmap. These analyses were performed by using R4.0.1 (29).

## Results

### Clinical Characteristics

From December 2019 to September 2020, a total of 53 patients were screened and 33 patients completed the whole study. Among these participants, 25 completed the pretreatment assessment and post-treatment assessment, of which 11 participants completed 1 month follow-up assessment; 8 participants completed the pretreatment assessment and 6 months follow-up assessment. We didn’t schedule the post-treatment assessment for these eight participants until the COVID-19 in China was well controlled, which was 6 months later after the end of treatment (**Figure S1**).

**Figure 1:**
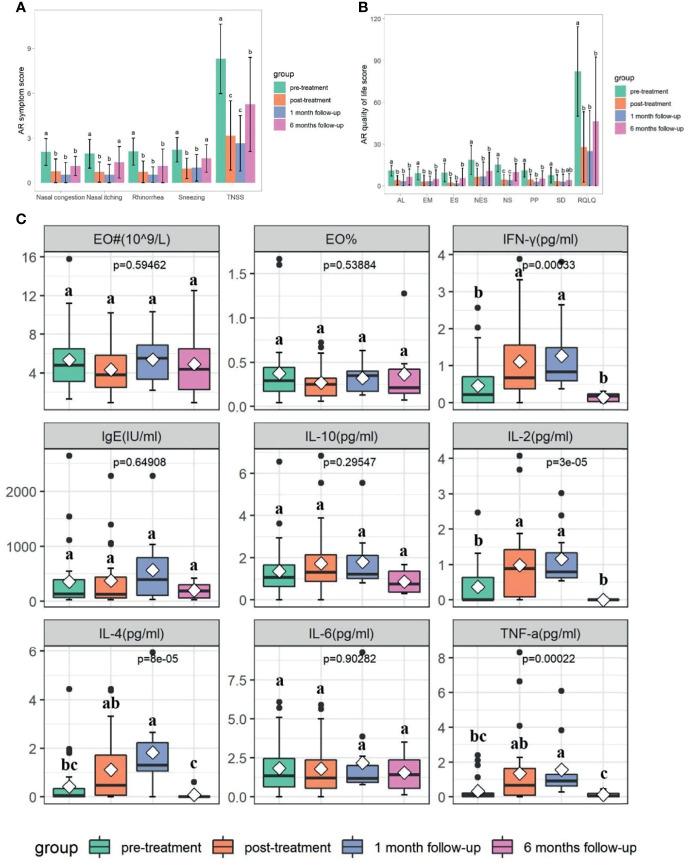
Changes in TNSS, RQLQ, EO%, EO counts, and cytokines at different time points based on Kruskal-Wallis test (p<0.05). **(A)** Changes in TNSS at different time points. **(B)** Changes in RQLQ at different time points. AL, Activity limitation; EM, Emotions; ES, Eye symptoms; NES, Non-eye/nasal symptoms; NS, Nasal symptom; PP, Practical problem; SD, Sleep disorders. **(C)** Changes in EO%, EO counts, and cytokines at different time points. EO# is the abbreviation of EO counts. Cytokines including IL-2, TNF-a, IFN-g, IL-4, IL-6, and IL-10. Letters (a, b, and c) indicate the difference grouping among different time points. Groups that do not share a common letter are significantly different.

A statistically significant decrease in TNSS and RQLQ was observed after medication, and such decrease lasted until the follow-up periods (*p*<0.05). The TNSS decreased from 8.30 ± 2.32 at week 0 to 1.79 ± 2.32 at week 10 to 2.64 ± 1.86 at week 14, and 5.25 ± 3.15 at week 34 (*p*<0.05) (**Table 1** and **Figure 1A**). The total score of RQLQ decreased from 82.21 ± 32.15 at week 0 to 18.03 ± 25.28 at week 10 to 25 ± 29.07 at week 14, and 46.38 ± 46.26 at week 34 (*p*<0.05) (**Table 1** and **Figure 1B**).

**Table 1:** Clinical characteristics of participants at different time points.

		Pretreatment	Post-treatment	1 month follow-up	6 months follow-up	*p* value
Subjects (n)		33	25	11	8	
Gender (Male/Female)		17/16	15/10	8/3	2/6	
Age (years)		27.30 ± 16.35	27.12 ± 16.71	23.64 ± 12.71	27.88 ± 16.23	
BMI		20.72 ± 4.45	20.43 ± 4.26	20.26 ± 4.19	21.29 ± 5.69	
SPT (n)	Dust mite−	3	3	2	0	
	Dust mite++	4	4	3	0	
	Dust mite+++	8	6	0	2	
	Dust mite++++	18	12	6	6	
TNSS		8.30 ± 2.32^a^	1.79 ± 2.32^c^	2.64 ± 1.86^c^	5.25 ± 3.15^b^	<0.001
	Rhinorrhea	2.09 ± 0.91^a^	0.58 ± 0.74^b^	0.55 ± 0.52^b^	1.13 ± 1.13^b^	<0.001
	Nasal congestion	2.06 ± 0.90^a^	0.67 ± 0.83^b^	0.55 ± 0.82^b^	1.13 ± 0.64^b^	<0.001
	Nasal itching	1.94 ± 0.97^a^	0.58 ± 0.68^b^	0.55 ± 0.69^b^	1.38 ± 1.06^a^	<0.001
	Sneezing	2.21 ± 0.82^a^	0.46 ± 0.68^b^	1.00 ± 0.89^b^	1.63 ± 0.92^a^	<0.001
RQLQ		82.21 ± 32.15^a^	18.03 ± 25.28^b^	25 ± 29.07^b^	46.38 ± 46.26^b^	<0.001
	Activity limitation (AL)	10.91 ± 3.80^a^	2.69 ± 3.41^b^	3.36 ± 4.67^b^	6.38 ± 5.76^b^	<0.001
	Sleep disorders (SD)	7.70 ± 5.70^a^	3.31 ± 4.72^b^	3.00 ± 5.29^b^	4.00 ± 4.87^ab^	<0.001
	Non-eye/nasal symptoms (NES)	18.61 ± 10.52^a^	4.34 ± 6.33^b^	6.82 ± 10.28^b^	10.63 ± 13.45^b^	<0.001
	Practical problem (PP)	11.21 ± 4.85^a^	2.82 ± 3.96^b^	2.82 ± 2.68^b^	5.13 ± 5.74^b^	<0.001
	Nasal symptom (NS)	15.12 ± 4.96^a^	2.54 ± 3.78^c^	4.09 ± 3.62^c^	9.75 ± 6.23^b^	<0.001
	Eye symptoms (ES)	9.61 ± 6.57^a^	2.41 ± 3.65^b^	1.73 ± 1.68^b^	5.50 ± 7.29^b^	<0.001
	Emotions (EM)	9.06 ± 4.80^a^	3.11 ± 4.75^b^	3.18 ± 3.76^b^	5.00 ± 6.44^b^	<0.001
Percentage of EO		5.27 ± 3.20^a^	4.35 ± 2.45^a^	5.39 ± 2.66^a^	4.90 ± 3.81^a^	0.54
EO counts (10^9^/L)		0.38 ± 0.36^a^	0.13 ± 0.18^a^	0.32 ± 0.16^a^	0.37 ± 0.40^a^	0.59
IgE(IU/ml)		355.22 ± 540.87^a^	379.42 ± 541.86^a^	566.57 ± 671.24^a^	198.46 ± 152.84^a^	0.65
Cytokines (pg/ml)	IL-2	0.37 ± 0.59^b^	0.80 ± 1.08^a^	1.16 ± 0.83^a^	0.00 ± 0.01^b^	<0.001
	IL-4	0.42 ± 0.91^bc^	1.02 ± 1.32^ab^	1.82 ± 1.55^a^	0.08 ± 0.21^c^	<0.001
	IL-6	1.83 ± 1.64^a^	1.37 ± 1.74^a^	2.18 ± 2.53^a^	1.55 ± 1.22^a^	0.9
	IL-10	1.36 ± 1.27^a^	1.10 ± 1.61^a^	1.81 ± 1.4^a^	0.87 ± 0.54^a^	0.3
	TNF-α	0.33 ± 0.61^bc^	1.36 ± 2.08^ab^	1.56 ± 1.79^a^	0.13 ± 0.18^c^	<0.001
	IFN-γ	0.46 ± 0.64^b^	0.86 ± 1.09^a^	1.27 ± 1.08^a^	0.15 ± 0.12^b^	<0.001
Safety evaluation	ALT (U/L)	23.85 ± 24.33	22.60 ± 23.49	21.82 ± 23.20	13.75 ± 5.23	0.68
	AST (U/L)	22.27 ± 8.96	22.12 ± 9.91	20.45 ± 9.38	18.75 ± 5.63	0.78
	BUN (mmol/L)	4.16 ± 1.04	4.13 ± 1.02	4.14 ± 0.88	4.53 ± 0.93	0.80
	CRE (umol/L)	60.94 ± 20.53	61.84 ± 17.02	55.09 ± 15.04	48.13 ± 9.39	0.15
	WBC (10^9/L)	6.40 ± 1.98	6.07 ± 1.50	6.14 ± 1.50	7.29 ± 2.60	0.68
	RBC (10^12/L)	4.78 ± 0.46	4.79 ± 0.42	4.85 ± 0.36	4.84 ± 0.81	0.93
	Hemoglobin (g/L)	129.79 ± 20.78	135.56 ± 13.21	136 ± 12.78	122.75 ± 7.11	0.06
	Platelet count(10^9/L)	234.32 ± 70.23	231.84 ± 46.07	231.18 ± 51.44	253.88 ± 102.00	0.97
	Hematocrit (%)	40.45 ± 3.97	41.16 ± 3.21	41.2 ± 3.32	37.95 ± 1.77	0.09

SPT is the abbreviation of skin prick test, which uses physiological saline as negative contrast, histamine as positive control, and dust mites drop as allergen. The result evaluation criteria were based on the rash caused by histamine, which means regardless of the size of the rash caused by histamine to pave +++, larger than the rash caused by histamine to pave the ++++, as large as the rash caused by histamine to pave the +++, smaller than the rash caused by histamine to pave the ++ or +, and negative for−. Letters (a, b, and c) indicate the difference grouping among different time points. Groups that do not share a common letter are significantly different.

There were no significant differences of tIgE, EO counts, percentage of EO, IL-6, and IL-10 at different time points (*p*>0.05) (**Table 1** and **Figure 1C**). A statistically significant increase in IL-2, IL-4, TNF-α, and IFN-γ was observed after medication, and such effect lasted until 1 month follow-up (*p*<0.05) (**Table 1** and **Figure 1C**), whilst these indicators decreased again at 6 months follow-up (*p*<0.05) (**Table 1** and **Figure 1C**).

### Dynamic Microbial Shifts After XQLD Treatment

**Figure 2:**
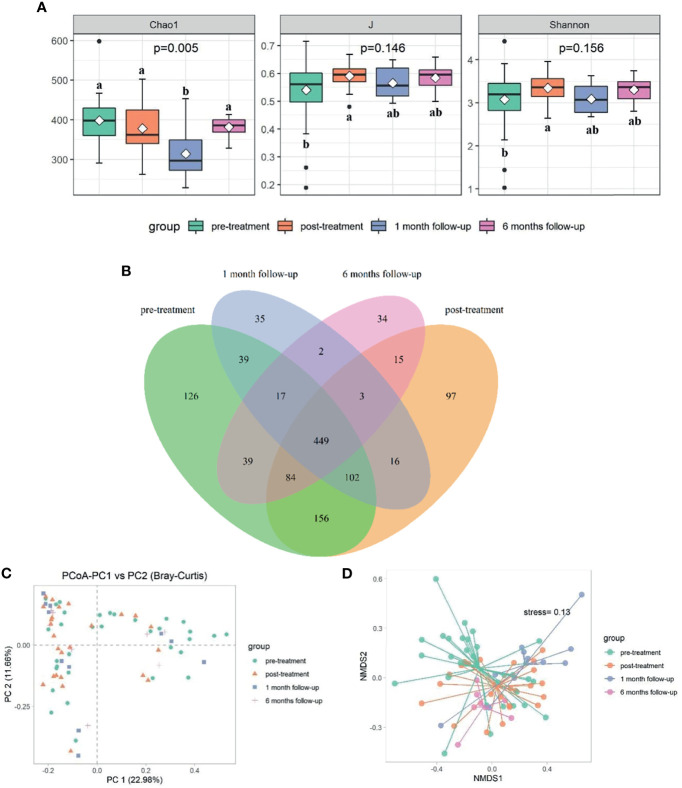
Comparison of bacterial diversity and composition at different time points (*p*<0.05). **(A)** The α diversity indexes comparison including Chao1, J, and Shannon. **(B)** Venn graph showing the shared and unique OTUs among different groups. The bacterial communities’ differences among different time points were reflected by PCoA plot **(C)** and NMDS **(D)**. Letters indicate the difference grouping among different time points. Groups that do not share a common letter are significantly different.

A total of 7,395,144 raw reads (80,000 reads/sample at pretreatment group; 103,262 reads/sample at post-treatment group; 104,817 reads/sample at 1 month follow-up group; 127,574 reads/sample at 6 months follow-up group), average of 70,796 OTU sequence/sample, and an average 374 OTUs were obtained from 77 samples (**Table S3**). There were significant differences in diversity indices Chao1 at different time points (*p*<0.05), while not for Shannon and Pielou’s evenness indices (J) (*p*>0.05) (**Figure 2A** and **Table S3**). Chao1 index significantly decreased after medication and continued until week 14 (*p*<0.05), while it rose again at week 34 (*p*<0.05) (**Figure 2A** and **Table S3**). Shared “universal” OTUs (OTU found in all groups) accounted for 36.99% of the total. Week 0 contained the most exclusive OTUs, while week 34 contained the least (**Figure 2B**).

**Figure 3:**
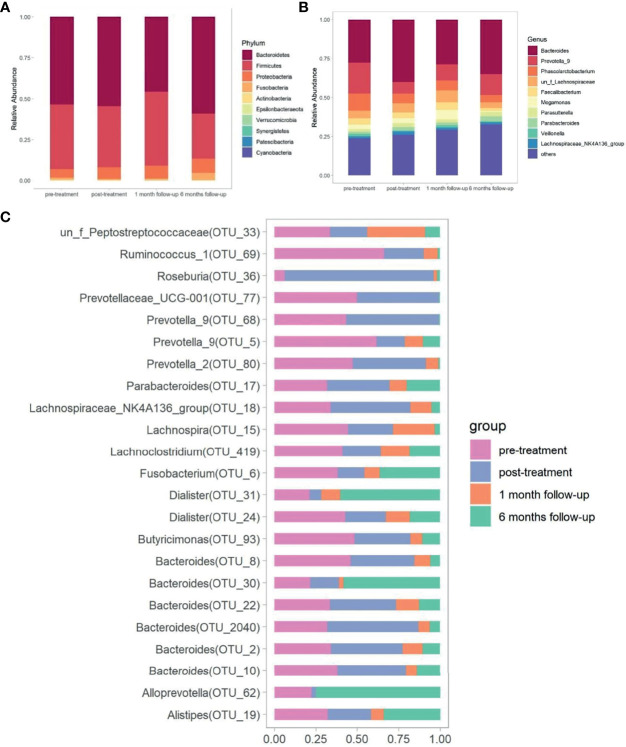
Comparison of gut microbiota at different time points. The bacterial phylum taxa **(A)** and genera **(B)** distribution pattern at different time points. **(C)** The distribution of significantly distinguished OTUs at different time points screened by Kruskal-Wallis test.

Significant differences in bacterial community structure at different time points were observed based on ANOSIM analyses (R^2^ = 0.094, *p*<0.05) and PERMANOVA analyses (R^2 ^= 0.062, *p*<0.05) and further graphed by Bray-Curtis distance based PCoA and NMDS plot (**Figures 2C, D**). Bacteroidetes (53.29%), Firmicutes (38.42%), and Proteobacteria (6.49%) were the dominated phyla taxa, occupying approximately 98.2% of the total (**Table S4**). The relative abundance of Bacteroidetes decreased to the lowest at week 14, while increased to the highest at week 34, which was the opposite condition in the abundance of Firmicutes (**Table S4** and **Figure 3A**). The relative abundance of Fusobacteria at week 14 was higher than that in other time points (**Table S4** and **Figure 3A**). Totally, 294 genera were observed, therein, *Bacteroides*, *Prevotella_*9, *Phascolarctobacterium*, un_f_Lachnospiraceae, *Faecalibacterium*, *Megamonas*, *Parasutterella*, *Parabacteroides*, *Veillonella*, Lachnospiraceae_NK4A136_group were the top 10 dominant genera, which added up to 73.71% (**Table S5** and **Figure 3B**). The relative abundance of *Bacteroides* increased at week 10, while decreased at week 14 and increased again at week 34 (**Figure 3B**). The relative abundance of *Prevotella_*9 decreased at week 10, while it increased again from week 14 to week 34 (**Figure 3B**). The relative abundance of *Phascolarctobacterium* continued to decline over time, which was the opposite condition in *Parabacteroides* (**Figure 3B**). The relative abundance of *Megamonas* significantly decreased at week 34 (**Figure 3B**).

The Kruskal-Wallis test was made to compare the significantly distinguished genera (*p*<0.05) at different time points. *Dialister* (OTU_31), *Roseburia* (OTU_36), *Alloprevotella* (OTU_62), *Alistipes* (OTU_19), *Bacteroides* (OTU_30), *Prevotella_*2 (OTU_80), *Prevotella_*9 (OTU_68), *Bacteroides* (OTU_2040), *Bacteroides* (OTU_10), and *Prevotella_9* (OTU_5) were the top 10 ranked distinguished genera (relative abundance≥0.1) (**Table S6** and **Figure 3C**). The relative abundance of *Dialister* (OTU_31) decreased to the lowest after treatment, but gradually increased over time (*p*<0.05). In contrast, the relative abundance of *Roseburia* (OTU_36), *Bacteroides* (OTU_2040), and *Bacteroides* (OTU_22) increased to the highest after treatment, but gradually decreased over time (*p*<0.05). The abundance of *Alloprevotella* (OTU_62) and *Bacteroides* (OTU_30) decreased until week 14, but raised again at week 34 (*p*<0.05). The lowest abundance of *Alistipes* (OTU_19) was observed at week 14 (*p*<0.05), and no significant difference was observed at other time points. The relative abundance of *Prevotella_*2 (OTU_80), *Bacteroides* (OTU_10), and *Prevotella_*9 (OTU_5) gradually decreased over time (*p*<0.05). The relative abundance of *Prevotella_*9 (OTU_68) increased at week 10 and then decreased at week 14 and increased again at week 34 (*p*<0.05) (**Table S6**).

### Association Between the Featured Bacterial Taxa and the Distinguished Pathways

**Figure 4:**
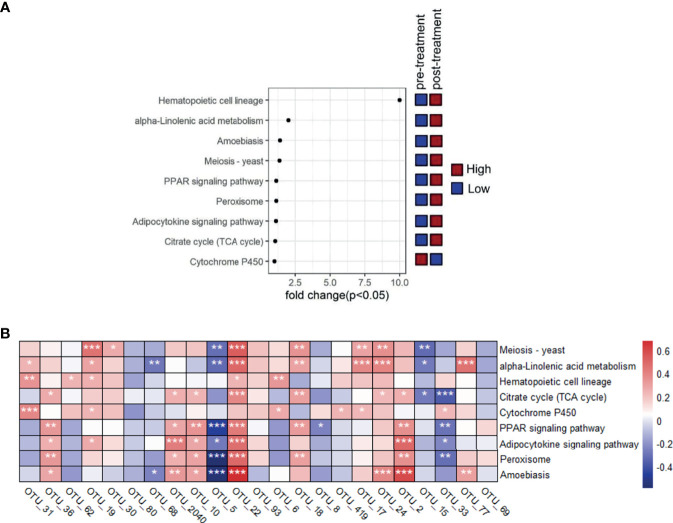
Comparison of metabolic pathway at different time points and the association between featured bacterial taxa and the distinguished pathways that were screened based on Kruskal-Wallis test (*p*<0.05). **(A)** Comparison of these distinguished pathways between pretreatment and post-treatment groups. **(B)** The correlationship between the nine distinguished pathways and the 23 significantly differed genera screened by Kruskal-Wallis test (*p*<0.05).

The functional pathway based on 16S rRNA gene were predicted by PICRUSt to obtain the pathway information that may be involved with AR condition. There were 272 detected pathways in KEGG level 3, and the top list included meiosis-yeast, alpha-linolenic acid metabolism, hematopoietic cell lineage, citrate cycle (TCA cycle), cytochrome P450, PPAR signaling pathway, adipocytokine signaling pathway, peroxisome, and amoebiasis. Then the Kruskal-Wallis test was made to compare the significantly distinguished features, and nine of them were screened out (*p*<0.05) (**Table S7**). The results showed that the pretreatment group had significant higher pathway associated with cytochrome P450 while lower meiosis-yeast, alpha-linolenic acid metabolism, hematopoietic cell lineage, citrate cycle (TCA cycle), PPAR signaling pathway, adipocytokine signaling pathway, peroxisome, and amoebiasis than the post-treatment group (*p*<0.05) (**Figure 4A**).

The correlationship between the nine distinguished pathway and the 23 significantly differed genera indicated that *Prevotella_*9 (OTU_5) was negatively related with meiosis-yeast, alpha-linolenic acid metabolism, PPAR signaling pathway, adipocytokine signaling pathway, peroxisome, and amoebiasis, which was the opposite condition for *Bacteroides* (OTU_22) (*p*<0.05). *Roseburia* (OTU_36), *Bacteroides* (OTU_2040), *Bacteroides* (OTU_2), and *Bacteroides* (OTU_10) were positively related with citrate cycle (TCA cycle), PPAR signaling pathway, adipocytokine signaling pathway, peroxisome, and amoebiasis (*p*<0.05) (**Figure 4B**).

### Key Phylotypes Responding to the XQLD Treatment in AR Patients

**Figure 5:**
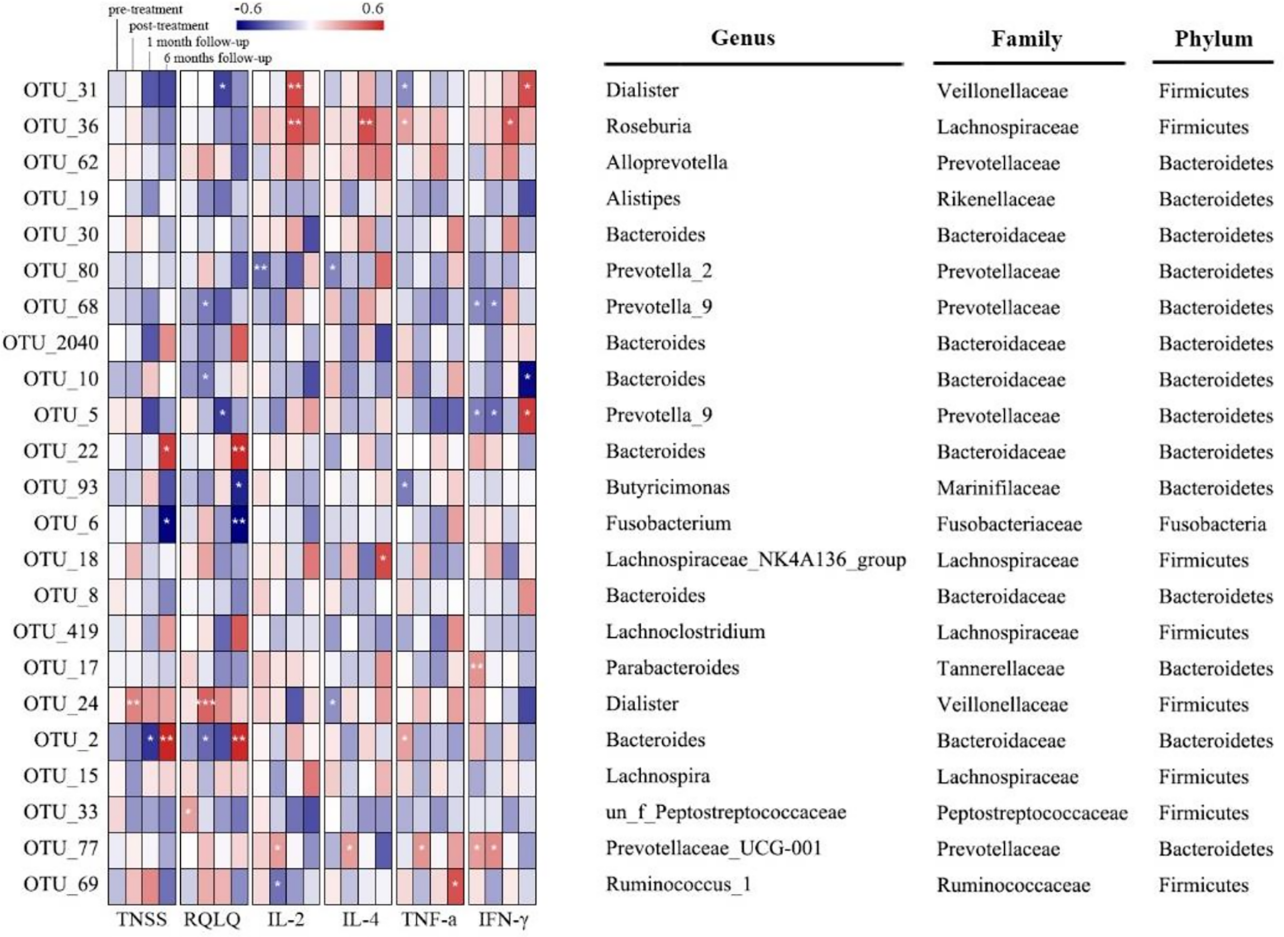
Correlation between distinguished bacterial taxa and TNSS, RQLQ, or cytokines based on Spearman’s correlation test (* means *p*<0.05; ** means *p*<0.01; *** means *p*<0.001.).

There was no significant correlation between Chao1 index and TNSS, RQLQ, or cytokines across different times (*p*>0.05) (**Table S8**). The significant associations between bacteria taxa, TNSS, RQLQ, and cytokines at different time points were analyzed by Spearman’s correlation test (**Figure 5** and **Table S9**). *Dialister* (OTU_31) was negatively related with RQLQ at week 14 as well as TNF-α at week 0, while positively related with IL-2 at week 14 and IFN-γ at week 34 (*p*<0.05). *Roseburia* (OTU_36) was positively related with IL-2, IL-4, and IFN-γ at week 14 as well as TNF-α at week 0 (*p*<0.05). *Prevotella_*2 (OTU_80) was negatively related with IL-2 and IL-4 at week 0 (*p*<0.05). *Prevotella_*9 (OTU_68) was negatively related with RQLQ and IFN-γ at week 10 (*p*<0.05). *Bacteroides* (OTU_10) was negatively related with RQLQ at week 10 and IFN-γ at week 34 (*p*<0.05). *Prevotella_*9 (OTU_5) was negatively related with RQLQ at week 14 and IFN-γ at week 0 and week 10, but positively related with IFN-γ at week 34 (*p*<0.05). *Bacteroides* (OTU_22) and *Bacteroides* (OTU_2) were positively related with TNSS score and RQLQ at week 34, which was opposite for *Fusobacterium* (OTU_6) (*p*<0.05). *Butyricimonas* (OTU_93) was negatively related with RQLQ at week 34 and IFN-γ at week 0 (*p*<0.05). *Dialister* (OTU_24) was positively related with TNSS score and RQLQ at week 10 and negatively correlated with IL-4 at week 0 (*p*<0.05). Prevotellaceae_UCG-001 (OTU_77) was positively related with IL-2, IL-4, TNF-α, and IFN-γ at week 10 (*p*<0.05). *Ruminococcus_*1 (OTU_69) was negatively related with IL-2 at week 10 and positively correlated with TNF-α at week 34 (*p*<0.05).

### Adverse Events

There were no instances of serious AEs observed or reported during the study. The only minor AEs observed during treatment were nausea (n=7), xerostomia (n=8), poor appetite (n=8), loose stools (n=2), and night sweating (n=1), but these events disappeared during the follow-up period. There were no significant differences at different time points in the safety evaluation (**Table 1**).

## Discussion

The current first-line therapy for AR was almost short-term effect. Once these medications are discontinued, most AR patients will relapse within a brief period (5). The unsatisfactory results of long-term AR treatment and the expressed need to develop these medication-sparing strategies within primary care created what has been termed an “effectiveness gap” in the management of this condition (30). There is a growing interest in the potential of TCM therapies to fill this gap. In this study, XQLD treatment provided significantly reduction in TNSS and RQLQ as compared with baseline (*p*<0.05), and such differences were maintained at a 6 months follow-up visit (*p*<0.05), indicating the long-term remission of AR after XQLD treatment.

The gut microbiota is hypothesized to have a critical role in allergic disease, including allergic rhinitis (AR) (2). Our previous study showed that *Bacteroides* is more abundant in healthy people as compared with AR patients, while Firmicutes is more abundant in AR patients (2). This study revealed that *Bacteroides* was substantially enriched after XQLD treatment, while Firmicutes was significantly decreased. Larger proportions of Bacteroidetes could be related to direct the development of Foxp3+ regulatory T cells (Tregs) and the release of immunomodulatory molecule such as polysaccharide A (31). However, larger proportions of Firmicutes have been reported to be associated with atopic disease, especially in the development of AR and asthma in early childhood (6, 32). The abundance of Bacteroidetes reached the highest and the abundance of Firmicutes reached the lowest at 6 months follow-up after XQLD treatment, which seems that the sustained decline of Firmicutes/Bacteroidetes (F/B) ratio until 6 months follow-up may contribute to the long-term remission of AR. Fusobacteria can elicit host proinflammatory response and possess virulence characteristics that promote their adhesiveness to host epithelial cells and to invade into epithelial cells, which was significantly increased at 6 months follow-up (33). The increase of pathogenic genera *Fusobacteria* at week 34 may explain why the TNSS and RQLQ at 6 months follow-up were still significantly lower than baseline, but higher than that of the end of treatment and 1 month follow-up (*p*<0.05), which may be in association with the prepathologic state.

Our previous study showed that the bacterial diversity in AR patients was significantly higher than that of healthy participants, which might be due to the expansion of bacteria taxa within Firmicutes (2). This study showed that the Chao1 index and the abundance of Firmicutes decreased after XQLD treatment and lasted until 1 month follow-up, while the Chao1 index increased again at 6 months follow-up, which may be related to the increase of pathogenic bacteria such as *Fusobacteria*.

The distinguished genera including *Dialister* (OTU_31), *Roseburia* (OTU_36), *Bacteroides* (OTU_22), *Bacteroides* (OTU_2040), and *Prevotella_*9 (OTU_5), which altered significantly across different time points after XQLD treatment, were closely related with the change of AR clinical indices. *Dialister*, *Roseburia*, and *Bacteroides* were reported to produce short-chain fatty acids (SCFAs) to bring benefits for the human host (34). *Dialister* was significantly enriched in 6 months follow-up as compared with the other three times, which was associated with a reduced risk of atopy (35). *Dialister* was negatively related with RQLQ at week 14 and positively related with anti-inflammatory factor IL-2 at week 14 as well as IFN-γ at week 34, which appear to protect against allergies (35). The highest abundance of *Roseburia* appeared at the end of XQLD treatment but decreased at the follow-up periods, which was positively related with anti-inflammatory factors. *Roseburia* likely plays a major role in maintaining gut health and immune defense, such as regulatory T-cell homeostasis, primarily through the production of butyrate (36). *Bacteroides* (OTU_22) and *Bacteroides* (OTU_2040) increased after XQLD treatment but decreased at the follow-up periods, of which *Bacteroides* (OTU_22) was negatively related with TNSS and RQLQ at week 10, while it was positively related with TNSS and RQLQ at week 34. *Prevotella_*9 produces sulfatases that degrade mucus polysaccharide, which could disrupt intestinal mucosal barrier and increase the production of pro-inflammatory cytokines in the body (37). The relative abundance of *Prevotella_*9 continued to decrease over time after XQLD treatment, which was negatively related with IFN-γ at week 0 and week 10, but positively related with IFN-γ at week 34. The increasing abundance of *Dialister*, *Roseburia*, *Bacteroides* (OTU_22), *and Bacteroides* (OTU_2040) together with the decreasing abundance of *Prevotella_9* may serve as microbial signature of long-term remission in AR after XQLD treatment. However, these findings need further verification for the true role of them in AR.

PPAR signaling pathway, which could be related to the reduction in inflammation and degradation of air pollutants, was higher in healthy people as compared with asthmatic patients (38). In this study, *Prevotella_*9 was negatively related with PPAR signaling pathway, which significantly reduced after XQLD treatment, while *Bacteroides* (OTU_22) and *Bacteroides* (OTU_2040) were positively related with PPAR signaling pathway and significantly enriched after XQLD treatment. The significantly higher expression of PPAR signaling pathway after XQLD treatment contributed to the reduction in inflammation of the nasal mucosa. Peroxisome proliferator–activated receptor γ (PPARγ) plays a role in the regulation of intestinal inflammation (39). Microbiota-activated PPAR-γ-signaling is a homeostatic pathway that prevents a dysbiotic expansion of potentially pathogenic genera by reducing the bioavailability of respiratory electron acceptors to Enterobacteriaceae in the lumen of the colon (39). SCFAs-producing bacteria *Roseburia*, *Bacteroides* (OTU_22), and *Bacteroides* (OTU_2040) were positively related with peroxisome, which was the opposite condition in *Prevotella_*9. Higher expression of peroxisome pathway reshaping the microbiota balance is helpful to the long-term remission of AR. Citrate cycle (TCA cycle) is a common metabolic pathway in aerobic organisms. In a 2,4,6-trinitrobenzenesulfonic acid (TNBS)-induced experimental colitis in rats, sulfasalazine (SASP), a sulfonamide antibiotic clinically used to treat IBD, was used to treat these experimental colitis rats. As a result, SASP not only restores the TNBS-induced gut dysbiosis but also modulates the dysregulated function of the TNBS-induced colitis, including an increased capacity for basic citrate cycle metabolism. This study also showed an upregulation of citrate cycle metabolism pathway after XQLD treatment (40). Both *Roseburia*, *Bacteroides* (OTU_22), and *Bacteroides* (OTU_2040) were positively correlated with citrate cycle metabolism. Therefore, the future research of probiotics which focuses on regulating the activity of PPAR signaling pathway, peroxisome, or citrate cycle (TCA cycle) can be used as a new target for the treatment of AR.

TCM formula has been developed and advocated for use in the treatment of many diseases for over 2,500 years in China. However, the complexities and unknown mechanisms of TCMs prevent the active chemical components from being identified. Our study suggests that gut microbiota might be involved in the effect of a widely used TCM formula, XQLD. This opens an avenue for identifying chemical components in TCMs, which can modulate gut microbiota structure as a potential mechanism for disease alleviation. Glycyrrhizin, paeoniflorin, ephedrine, and catechin were identified as the four most abundant chemical components in XQLD (41). Glycyrrhizin selectively induces mature T lymphocyte apoptosis, modulates quantity and function of lymphocytes, and corrects disorder of T lymphocytic subgroups in circular blood. Besides, glycyrrhizin also could inhibit the production of inflammatory cytokines and inflammatory mediator, and antagonizes inflammatory reaction of inflammatory mediator (42). Additionally, glycyrrhizin have been reported to prevent high-fat-diet (HFD)-enhanced premetastatic niche formation of tumor and metastasis by gut microbiota, implying that glycyrrhizin could be one of the major active ingredients in XQLD that modulated the gut microbiota in our study (43). Paeoniflorin has anti-inflammatory, analgesic, and immunomodulatory effects, but its oral absorption rate is very low. Oral administration of paeoniflorin plays its physiological role and requires the catalysis of β-glucosidase and esterase secreted by intestinal bacteria (44). Ephedrine demonstrated antiallergic properties together with the inflammation-lowering effects in OVA-induced asthma rats (45). However, no study has reported the impact of ephedrine on gut microbiota. Catechin can effectively alleviate Th2 type inflammation in OVA-induced asthma rats, and the new catechin derivatives displayed anti-inflammatory effects in a hapten-induced mouse contact dermatitis model (46, 47). Catechin can significantly increase the bacterial diversity and the abundance of butyrate- and acetate-producing bacteria in HFD-induced rats (48). These results indicate that modulation of gut microbiota by chemical components, such as glycyrrhizin, paeoniflorin, ephedrine, and catechin, might be involved in improving AR by XQLD, suggesting that TCMs may serve as a new source for drug leads in gut microbiota-targeted AR long-term management.

There were several limitations to this study. Firstly, we included a limited number of subjects in a cases series study, which reminds of us that more samples included in a RCT should be considered to make more robust conclusion. Secondly, each time point’s bacterial metabolites should be detected because metabolites of gut microbiota may be a bridge to link the host and bacteria. Thirdly, each week’s fecal sample should be collected for 16S rRNA test so that we can judge whether the gut microbiota alterations occurred before or after the significant improvement of AR symptoms. Overall, further studies of larger sample size, RCT, host-microbiome interaction are needed to validate these findings, which will make us clearly understand the precise roles of gut microbiota in the sustained remission of XQLD treatment for AR.

In summary, AR patients with a microbiota signature high in *Dialister* (OTU_31), *Roseburia* (OTU_36), *Bacteroides* (OTU_22), and *Bacteroides* (OTU_2040) and low in *Prevotella_*9 (OTU_5) could be predictive of good sustained response to XQLD treatment. SCFA producers mainly dominate this favorable signature, and durable response is associated with a restoration of the significantly high expression of PPAR signaling pathway, peroxisome, or citrate cycle (TCA cycle) found in AR patients. The relative high abundance of Fusobacteria can be used as a biomarker species predictive of a prepathologic state in AR.

## Data Availability Statement

The datasets presented in this study can be found in online repositories. The names of the repository/repositories and accession number(s) can be found in the article/**Supplementary Material**.

## Ethics Statement

The studies involving human participants were reviewed and approved by the Ethics Committee of Xiamen hospital of Traditional Chinese Medicine (Permit Number ID: 2019-K020-01). Written informed consent to participate in this study was provided by the participants’ legal guardian/next of kin.

## Author Contributions

Conceived and designed the study: LZ, WW, YD and WL. Participated in investigation: LT, BY, WW, YD, and JX. Performed formal analysis: LZ, WL, and YW. Collected the resources: LZ, LT, BY, CL, ZC, and JX. Curated the data: LZ, YW, ZC, CL, and WL. Wrote the manuscript: LZ, YW, and WL. Supervised the study: LZ, ZC, and YW. All authors approved the final version of the manuscript and agreed for publication once accepted. All authors contributed to the article and approved the submitted version.

## Conflict of Interest

The authors declare that the research was conducted in the absence of any commercial or financial relationships that could be construed as a potential conflict of interest.

## Publisher’s Note

All claims expressed in this article are solely those of the authors and do not necessarily represent those of their affiliated organizations, or those of the publisher, the editors and the reviewers. Any product that may be evaluated in this article, or claim that may be made by its manufacturer, is not guaranteed or endorsed by the publisher.
