# Supplementary figures and images for: Dynamic Microbial Shifts and Signatures of Long-Term Remission in Allergic Rhinitis After an Herbal Formula Treatment
Source: Front Immunol. 2021 Oct 22;12:774966. doi: 10.3389/fimmu.2021.774966 (PMC8569905; doi:10.3389/fimmu.2021.774966)

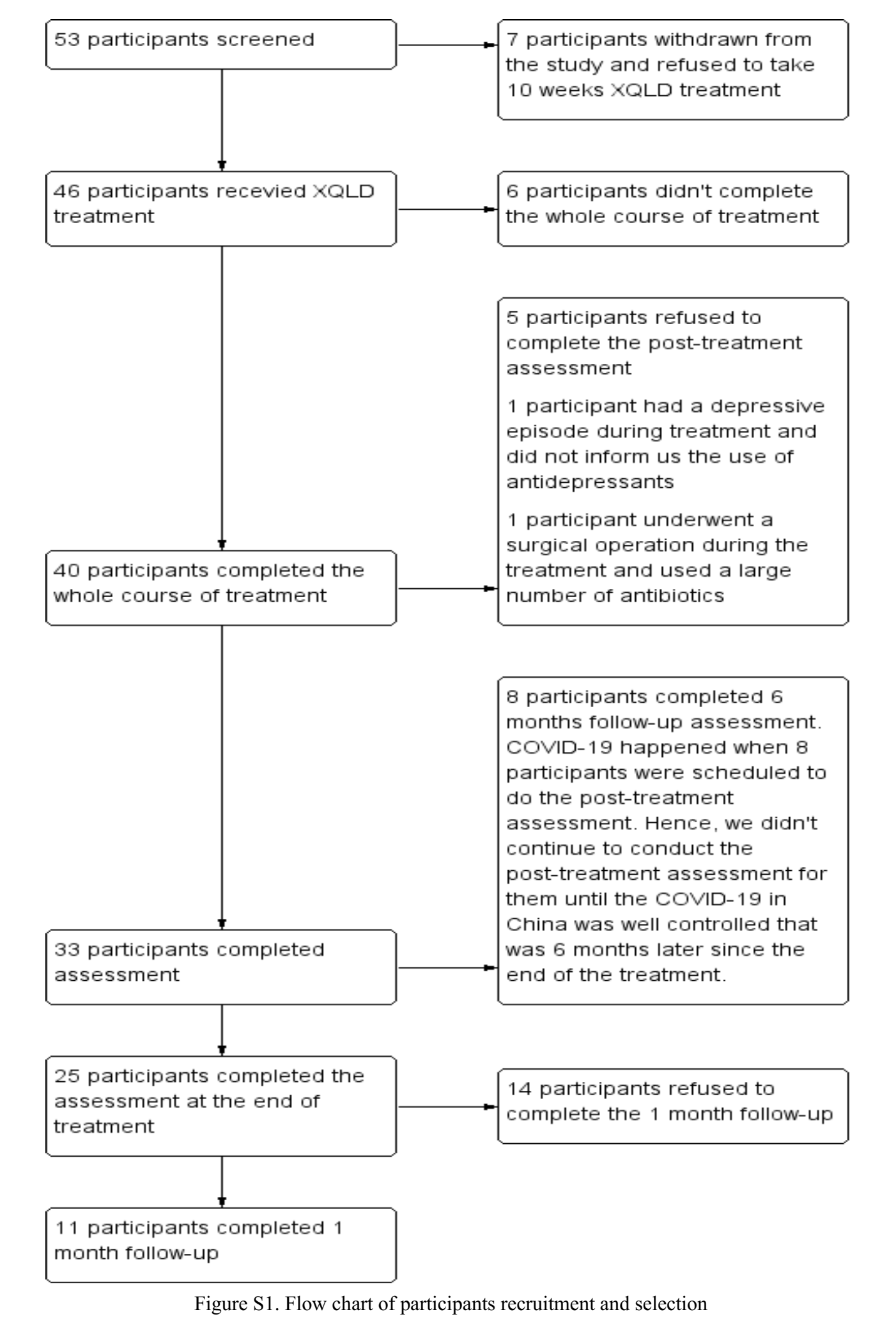

Supplement: Supplementary file 2 [file Image_1.jpeg]
